# Biomembrane-Modified Biomimetic Nanodrug Delivery Systems: Frontier Platforms for Cardiovascular Disease Treatment

**DOI:** 10.3390/biom14080960

**Published:** 2024-08-07

**Authors:** Yunan Gu, Lixin Du, Yuxin Wu, Juan Qin, Xiang Gu, Zhihua Guo, Ya Li

**Affiliations:** 1 School of Pharmacy, Hunan University of Chinese Medicine, Changsha 410208, China; 202104010106@stu.hnucm.edu.cn (Y.G.); 20223723@stu.hnucm.edu.cn (L.D.); 202104010307@stu.hnucm.edu.cn (Y.W.); 202104010225@stu.hnucm.edu.cn (J.Q.); 202204010342@stu.hnucm.edu.cn (X.G.); 2 School of Chinese Medicine, Hunan University of Chinese Medicine, Changsha 410208, China; 004294@hnucm.edu.cn

**Keywords:** biomembrane, targeting, biomimetic nanodrug delivery systems, cardiovascular diseases

## Abstract

Cardiovascular diseases (CVDs) are one of the leading causes of death worldwide. Despite significant advances in current drug therapies, issues such as poor drug targeting and severe side effects persist. In recent years, nanomedicine has been extensively applied in the research and treatment of CVDs. Among these, biomembrane-modified biomimetic nanodrug delivery systems (BNDSs) have emerged as a research focus due to their unique biocompatibility and efficient drug delivery capabilities. By modifying with biological membranes, BNDSs can effectively reduce recognition and clearance by the immune system, enhance biocompatibility and circulation time in vivo, and improve drug targeting. This review first provides an overview of the classification and pathological mechanisms of CVDs, then systematically summarizes the research progress of BNDSs in the treatment of CVDs, discussing their design principles, functional characteristics, and clinical application potential. Finally, it highlights the issues and challenges faced in the clinical translation of BNDSs.

## 1. Introduction

Cardiovascular diseases (CVDs) are one of the leading causes of death worldwide, posing a significant threat to human health [1,2]. According to the World Health Organization (WHO), approximately 17.7 million people die from CVDs each year, accounting for 31% of all global deaths [3]. CVDs not only result in high mortality rates but also severely impact the quality of life of patients, imposing substantial economic burdens on families and society. The pathogenesis of CVDs is complex and typically associated with factors such as hypertension, hyperlipidemia, diabetes, and unhealthy lifestyles [4,5]. With the accelerating global aging process and changes in lifestyle, the incidence of CVDs is on the rise [6]. Current treatments for CVDs primarily include general therapy, surgical intervention, and pharmacological treatment [7]. General therapy mainly involves lifestyle improvements to alleviate the occurrence or symptoms of CVDs [8]. Surgical treatment [9] addresses the pathological site through interventional means but has strict indications and causes significant trauma to the body. Pharmacological treatment [10] is the most common and important approach for CVDs, with wide clinical application and significant efficacy. Despite substantial progress in the diagnosis and treatment of CVDs in modern medicine, traditional drug therapy remains limited due to the complexity and heterogeneity of the disease. Issues such as insufficient specificity of drugs, systemic toxicity, and difficulties in effectively delivering drugs to the lesion sites severely constrain therapeutic outcomes [11,12]. Therefore, there is an urgent need to develop an effective and convenient drug treatment method.

To enhance the therapeutic efficacy of CVDs, scientists continuously explore novel treatment methods and drug delivery systems. Among these, nanotechnology has emerged as a focal point of research due to its unique advantages in drug delivery. Nanodrug delivery systems (NDSs) can significantly improve drug bioavailability and targeting capabilities [13], reducing nonspecific distribution and side effects within the body [14]. NDSs can facilitate drug passage across biological barriers, such as the blood–brain barrier and vascular endothelial cell gaps, thereby achieving efficient drug delivery to diseased sites [15]. Additionally, NDSs possess high surface area and multifunctional surfaces, allowing for surface modifications to achieve targeted delivery to specific targets, enhancing drug concentration at pathological sites and improving therapeutic outcomes while minimizing nonspecific distribution and side effects in healthy tissues [16,17]. Furthermore, NDSs can achieve controlled release and long-term drug delivery, maintaining stable drug concentrations in the body, reducing dosing frequency, and improving patient compliance [18,19]. However, traditional NDSs face complex clearance and excretion mechanisms in vivo and are easily recognized and cleared by the immune system, leading to low drug delivery efficiency [20,21].

To address this issue, a novel nanodrug delivery platform has emerged in recent years—biomembrane-modified biomimetic nanodrug delivery systems (BNDSs). By modifying the surface of nanoparticles with biological membranes, such as erythrocyte membranes and platelet membranes, this design effectively reduces recognition and clearance by the immune system, enhancing biocompatibility and circulation time of the drugs in the body, thereby improving therapeutic efficacy [22,23]. On one hand, biological membranes inherently possess certain targeting properties; after modification, the drugs are enveloped in a “protective coat”, significantly enhancing the targeting capability of drug delivery and ensuring more precise delivery of drugs to diseased sites [24]. On the other hand, BNDSs not only improve the stability of drugs but also enable controlled release functions. By combining various functional materials and drugs, they can achieve multifunctional therapeutic effects [25].

This innovative design offers new hope and research directions for the treatment of CVDs, showcasing broad application prospects. This paper provides a brief overview of the classification and pathological mechanisms of CVDs, elucidates the construction principles and types of BNDSs, and systematically reviews the research progress of BNDSs in the diagnosis and treatment of CVDs. Finally, it highlights the challenges and issues in the clinical translation of BNDS and proposes some perspectives for future research.

## 2. Overview of CVD Classification and Pathological Mechanisms

**Figure 1:**
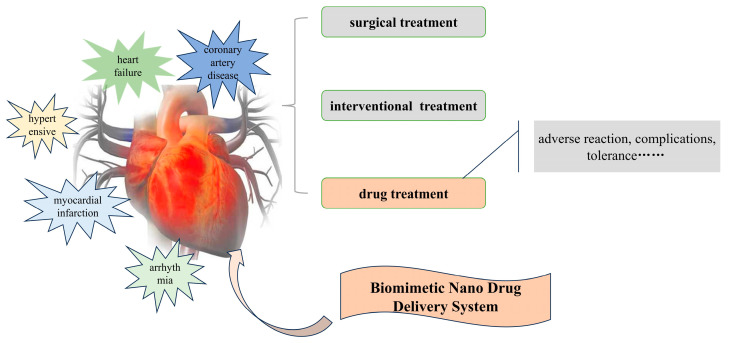
Schematic diagram of CVD classification and treatment.

CVDs, also known as circulatory system diseases, are a collective term for heart, vascular, and peripheral vascular diseases [26]. CVDs include coronary artery disease [27], myocardial infarction [28], hypertension [29], arrhythmias [30], and heart failure [31]. These diseases are closely related, and a schematic diagram of CVD classification and related treatments is shown in Figure 1. When vascular lesions occur, they can easily lead to coronary artery disease, reducing blood flow to the heart and causing myocardial infarction [32]. Hypertension can further exacerbate the risk of coronary artery disease and myocardial infarction [33]. Structural and functional changes in the heart after a myocardial infarction can lead to heart failure [34], while arrhythmias can result from coronary artery disease and heart failure due to myocardial hypoxia and electrophysiological abnormalities [35].

### 2.1. Coronary Artery Disease

Coronary artery disease is a type of CVD caused by the formation of plaques within the coronary arteries, leading to insufficient blood supply to the myocardium. Its core pathological mechanism is atherosclerosis [36]. Endothelial cell injury and dysfunction are early markers of coronary artery disease. When vascular endothelial cells are damaged, low-density lipoprotein (LDL) particles infiltrate the arterial wall and undergo oxidation to form oxidized low-density lipoprotein (ox-LDL). The oxidized LDL particles attract monocytes into the arterial wall, which differentiate into macrophages. These macrophages phagocytose ox-LDL, transforming into foam cells and forming fatty streaks that narrow the vascular lumen [37]. When coronary artery narrowing reaches a critical level, the myocardium requires more blood flow during physical exertion or emotional stress. However, due to the inability of the coronary arteries to sufficiently dilate, myocardial ischemia occurs, impairing the microcirculation of the coronary arteries and manifesting as angina [38].

### 2.2. Myocardial Infarction

Myocardial infarction refers to the pathological process of myocardial ischemia and necrosis caused by the sudden interruption of coronary blood flow [39]. On one hand, myocardial infarction is related to coronary artery lesions caused by coronary artery disease [40]. When a plaque in the coronary artery ruptures or erodes, platelets rapidly adhere to the exposed plaque surface and activate, releasing various procoagulant substances such as ADP, leading to microthrombus formation in distal vessels, further exacerbating myocardial ischemia. On the other hand, ischemic necrosis of cardiomyocytes is central to myocardial infarction, with fibroblasts playing a role in the repair and scar formation post-infarction. Necrosis of cardiomyocytes can trigger systemic or localized inflammatory responses, leading to infiltration of neutrophils and monocytes into the damaged area. These cells clear necrotic tissue and release various inflammatory mediators, potentially expanding the extent of damage and resulting in ventricular remodeling and dysfunction [41].

### 2.3. Hypertension

Hypertension is a chronic condition characterized by persistently elevated blood pressure, with a complex pathophysiology involving multiple physiological and pathological processes [42]. In addition to the combined influence of genetic and environmental factors, hypertension is typically associated with the constriction of arterioles and microvessels. Endothelial cell dysfunction is one of the key mechanisms of hypertension [43,44]. Vascular smooth muscle cell proliferation and hypertrophy are key characteristics of hypertension, while endothelial cell dysfunction leads to vasoconstriction and elevated blood pressure. Endothelial cells lose their normal vasodilatory function, resulting in increased release of vasoconstrictors (such as endothelin, ET) and decreased release of vasodilators (such as nitric oxide, NO), leading to vascular constriction and increased peripheral vascular resistance [45]. Furthermore, the production of angiotensin II (Ang II) is increased under the action of angiotensin-converting enzyme (ACE) [46], which stimulates aldosterone secretion, increases sodium and water reabsorption, and further elevates blood pressure.

### 2.4. Arrhythmia

Arrhythmia refers to abnormal heart rhythms, including tachycardia, bradycardia, or irregular heartbeats. Its pathological mechanisms involve multiple aspects, such as cardiac electrophysiology, ion channel function, and neural regulation [47]. Electrophysiological abnormalities in cardiomyocytes and rhythm disturbances in pacemaker cells are major causes of arrhythmia. When myocardial tissue is diseased, particularly in conditions such as coronary artery disease and heart failure, electrophysiological abnormalities are more likely to occur. Reentry is the most common mechanism of arrhythmia, referring to the cyclic propagation of electrical signals within the heart, triggering rapid and repetitive cardiac excitation. These electrophysiological abnormalities can induce dysfunction in the ion channels on the cardiomyocyte membrane, such as Na channels, K channels, and Ca channels, leading to the occurrence of arrhythmia [48].

### 2.5. Heart Failure

Heart failure is the terminal stage of heart disease, characterized by impaired systolic or diastolic function of the heart, rendering it unable to meet the body’s demands for oxygen and blood. Nearly all CVDs ultimately lead to the development of heart failure [49]. Loss of function and apoptosis of cardiomyocytes are central to heart failure, fibroblasts play a role in cardiac remodeling, and endothelial cell dysfunction affects cardiac blood supply. Heart failure typically begins with structural changes in the heart; ischemia and necrosis of the myocardium cause ventricular remodeling and dysfunction, leading to damaged myocardial cells and increased energy consumption, thereby imposing greater stress on the heart [50]. Another pathological mechanism of heart failure is often associated with inflammation and oxidative stress. Following immune dysregulation in heart failure, inflammatory cells in the body release inflammatory cytokines (such as TNF-α, IL-6, NF-κB), which drive the progression and worsening of heart failure [51]. Oxidative stress is characterized by the direct damage to myocardial cells by reactive oxygen species, resulting in membrane rupture, organelle dysfunction, and ultimately, a decline in cardiac function [52].

## 3. Construction Principles of BNDSs

### 3.1. Source of Biomembranes

BNDSs are drug delivery systems prepared using biomimetic biomaterials such as cell membranes, extracellular vesicles, hybrid membranes, etc., as drug carriers [53]. Cell membranes encompass materials such as erythrocyte membranes, macrophage membranes, platelet membranes, stem cell membranes, and tumor cell membranes. The core of BNDSs lies in enhancing drug delivery targeting and biocompatibility through modification. Given the broad sources of biological membranes, BNDSs offer a rich array of choices for nanodrug delivery [54].

Among the cell membranes used to construct BNDSs, erythrocyte membranes are the most widely applied [55]. Erythrocytes, being the most abundant cells in human blood, are easy to obtain. They are biconcave with a diameter of 6–8 μm and a thickness of 2 μm, lacking nuclei and organelles, which simplifies their intracellular environment and provides a large surface area for drug loading [56]. Erythrocyte membranes exhibit high biocompatibility, do not trigger immune rejection, and have a long circulation time in the body, enhancing drug bioavailability and circulation duration [57]. Nanocarriers modified with macrophage membranes can modulate immune responses, evade rapid clearance by the immune system, extend drug circulation time, and naturally chemotax to inflammatory sites and tumor microenvironments [58]. Additionally, platelet membranes possess innate inflammation-targeting properties [59], allowing precise localization to damaged vessels or inflamed areas. Stem cell membranes [60] have low immunogenicity and excellent migration capabilities, making them suitable for constructing multifunctional nanocarriers for myocardial repair and regenerative medicine, promoting tissue regeneration and functional recovery. Tumor cell membranes, with their specific antigen characteristics, can target and bind to tumor cells, increasing drug accumulation at tumor sites and showing significant promise in cancer therapy [61]. Despite the advantages of biomembranes in drug delivery, there are also some drawbacks. A summary of the pros and cons of various biomembranes is presented in Table 1.

### 3.2. Biomimetic Principles of Biomembranes

BNDSs exhibit superior intelligent delivery performance by mimicking biological structures in chemical, physical, and morphological aspects, excelling in inert rejection, overcoming barriers, and active functions. The biomimetic principles of biomembranes in BNDSs involve using natural cell membranes or synthetic membrane structures as carriers to replicate and leverage their specific functions and properties in vivo, achieving targeted drug delivery, biocompatibility, and stability [69,70]. Biomembranes feature complex structures, including phospholipid bilayers, protein channels, and receptors, which provide inherent targeting capabilities. For instance, platelet membranes can adhere to damaged blood vessel walls [71], and macrophage membranes can localize to inflammation sites, enabling localized drug delivery [72].

Self-assembly is a crucial synthesis principle in BNDSs, where molecules aggregate via non-covalent bonds to form various nanostructures and functions, regulated by external stimuli or the environment. This method is common in biomimetic cell membrane technology, where adjusting lipid molecules’ properties and environmental conditions enables the spontaneous formation of artificial lipid bilayers [73]. In solution, drug molecules can spontaneously form micelles or micelle-like nanoclusters, with hydrophilic moieties exposed to the surrounding solvent on the outside and hydrophobic parts “fixed” on the inside, resulting in self-assembly.

The selection and functional modification of biomembranes are vital in BNDSs. Scientists extract and purify membranes from different cell types and encapsulate them on the surface of nanodrugs, retaining the natural properties of the biomembranes and conferring targeting and functional characteristics to the nanodrugs [74]. Additionally, synthetic biomimetic membranes play a significant role in drug delivery systems, designed and controlled precisely to achieve more accurate drug delivery and release [75]. Functional modifications allow synthetic biomimetic membranes to mimic natural membrane functions, such as recognizing target molecules and intracellular drug release mechanisms [76]. Compared to traditional nanocarriers, biomembranes offer excellent biocompatibility and low immunogenicity, effectively avoiding immune system recognition and clearance, extending circulation time, and enhancing bioavailability, making them safer and more reliable in drug delivery systems [77,78].

## 4. Extraction of Biomembranes and Construction of BNDSs

### 4.1. Extraction of Biomembranes

The extraction of biomembranes is a critical step in BNDS construction, directly impacting the quality and functionality of the carriers. The process typically involves isolating and purifying membranes from cells or tissues to ensure they possess the required biological activity and characteristics. Common extraction methods and their characteristics are shown in Table 2.

### 4.2. Integration of Biomembranes with Drugs

#### 4.2.1. Drug Adsorption on Biomembranes

Drug molecules can adhere to the surface or outer layer of biomembranes through physical forces such as electrostatic attraction, hydrophobic interactions, or hydrogen bonds in a non-covalent manner [83]. While simple and feasible, this method limits drug stability and release. Alternatively, specific functional groups on the biomembrane surface (e.g., hydroxyl, amino, carboxyl groups) can form covalent bonds with functional groups on drug molecules (e.g., ester bonds, amide bonds, conjugates) [84,85]. This approach typically requires pre-designed linking compounds to ensure robust attachment of drugs to biomembranes. Researchers have fused platelet membranes and stem cell-derived extracellular vesicles using a porous polycarbonate membrane for the treatment of atherosclerosis. Nanoparticle tracking analysis indicated excellent stability of the drug delivery system, and in vivo results in ApoE^−/−^ mouse models showed significant reductions in lipid deposition and necrotic core area [86]. Additionally, another study developed macrophage membrane-modified MnO2 fenugreek nanoparticles. These drug-loaded nanoparticles, upon adhesion to macrophage membranes, can recognize cell surface molecules overexpressed on damaged vascular endothelium, reversing the pro-inflammatory microenvironment to mitigate ischemia-reperfusion injury [87].

#### 4.2.2. Drug Encapsulation in Biomembranes

Drug molecules are encapsulated within the lipid bilayer structure of biomembranes, utilizing its self-assembly and encapsulation capabilities [88]. The lipophilic regions of the membrane interact with drug molecules through affinity with lipid components and aqueous solubility interactions, thereby extending drug release [89]. Certain drug molecules also specifically bind to proteins or peptides on the biomembrane, leveraging their high specificity and affinity for encapsulation [90]. This approach enhances drug stability and enables targeted delivery to specific targets. Yang et al. [91] developed a hybrid membrane-coated biomimetic nanosystem (PM/RM@PLGA@P/R) using poly(lactic-co-glycolic acid) (PLGA) as a carrier, camouflaged with erythrocyte membrane (RM) and platelet membrane (PM), achieving targeted delivery of ginsenoside Rg1 and perfluorohexane (PFH). This nanoparticle cleverly combines antioxidant, anti-inflammatory, and anticoagulant capabilities of drugs, demonstrating superior in vitro performance in ROS scavenging, anticoagulant activity, and immune evasion compared to nanoparticles without hybrid membrane coating.

### 4.3. Construction of BNDSs

**Figure 2:**
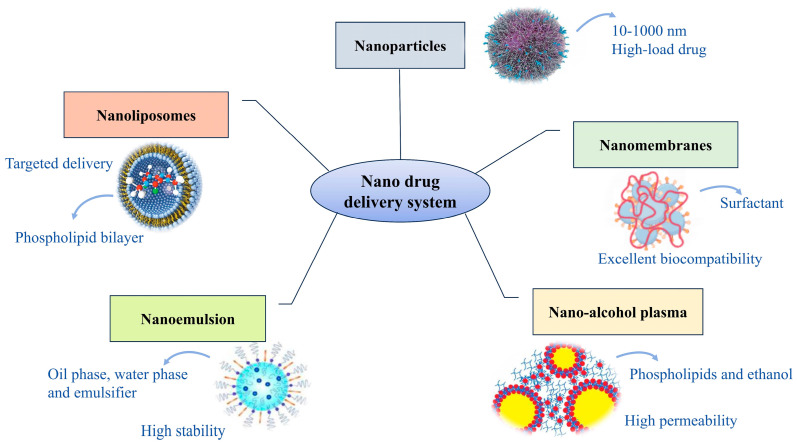
Classification diagram of BNDSs.

A BNDS is not a single drug formulation but an advanced drug delivery platform comprising various formulations. We summarize research on BNDSs for treating CVDs, which currently include nanoparticles, nanoliposomes, nanomenbranes, nanoemulsion, and nano-alcohol plasma [92], as shown in Figure 2. Characteristics and applications of these formulations are detailed in Table 3.

## 5. Applications of BNDSs in the Treatment of CVDs

### 5.1. Drug Delivery, Release, and Targeted Therapy

#### 5.1.1. Erythrocyte Membranes

**Figure 3:**
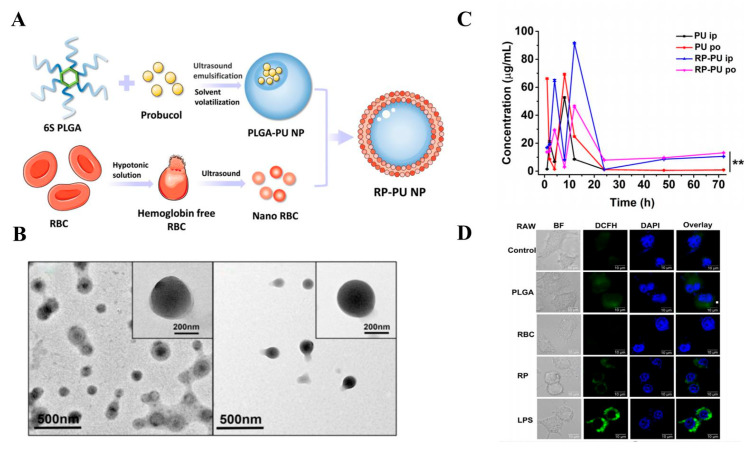
(**A**) Schematic representation of RP-PU NP formation. (**B**) Transmission electron microscopy results of RP-PU NPs. ** *p* < 0.01 vs. RU po. (**C**) Pharmacokinetic study demonstrating slow release of RP-PU NPs after 24 h. (**D**) RP-PU NPs reducing ROS levels in RAW cells. Abbreviations: DCFH, 2′,7′-Dichlorofluorescin; DAPI, 4′,6-Diamidino-2-phenylindole dihydrochloride. Adapted with permission from ref. [98]. Copyright © 2022 Elsevier Inc. All rights reserved.

Erythrocyte membranes are widely applied in constructing BNDSs, showing significant breakthroughs in drug delivery and treatment of CVDs. Liang et al. [98] extracted erythrocyte membranes from C57BL/6J mice, which were mixed with PLGA, the drug probucol (PU), and 2 mL dichloromethane, followed by ultrasonication and centrifugation for 4 h to obtain biomimetic nanoparticles (Figure 3A). Transmission electron microscopy revealed a double-membrane structure with successful encapsulation of PU NPs within erythrocyte membranes (Figure 3B). Pharmacokinetic studies demonstrated that the nanoparticle group (RP-PU NPs) achieved peak drug concentration in blood at 12 h and exhibited sustained release thereafter (Figure 3C). Inflammation induced by myocardial injury typically increases intracellular ROS levels; therefore, they also assessed ROS levels in RAW cells, showing that RP-PU NPs had superior ROS scavenging effects (Figure 3D). HE staining demonstrated effective improvement in atherosclerotic plaques in the RP-PU NPs group, with restored vascular morphology and reduced collagen fiber content, indicating that RP-PU NPs could reduce tissue fibrosis and protect cardiac function.

To enhance the responsiveness of drug delivery systems, researchers have developed a shear stress and ROS microenvironment-specific BNDS; self-assembled simvastatin (SA), erythrocyte membranes, and cross-linked polyethyleneimine nanoparticles (PEI) form SA PEI@RBCs for treating atherosclerosis. Shear stress model experiments demonstrated that SA PEI@RBCs respond effectively to high shear stress, enhancing SA PEI accumulation and therapeutic efficiency. Both in vitro and in vivo studies confirmed that SA PEI@RBCs exhibit superior safety and therapeutic efficacy compared to SA PEI and free SA. Thus, shaping SA PEI@RBCs with dual sensitivity to ROS and shear stress represents an effective strategy and treatment approach for facilitating delivery into plaques [99].

#### 5.1.2. Macrophage Membranes

**Figure 4:**
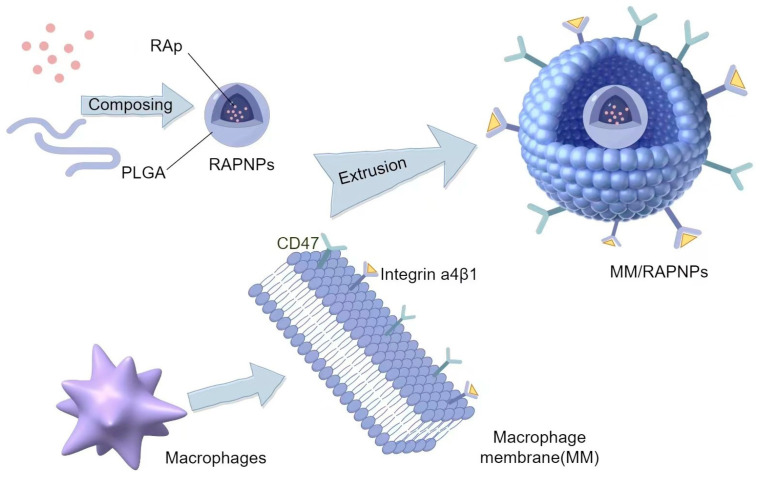
Schematic diagram of MM/RAPNP preparation.

Macrophages are highly functional white blood cells and major cellular effectors in inflammation and tissue repair. Therefore, nanodrug delivery systems coated with macrophage membranes often exhibit enhanced targeting effects for inflammatory diseases [100]. Wang et al. [101] used a direct extrusion method to prepare functionalized MM/RAPNPs by mixing macrophage vesicles with PLGA nanoparticles. In vitro studies demonstrated their good biocompatibility, minimal cytotoxicity, and ability to inhibit macrophage phagocytosis. They also exhibited targeted binding to endothelial cells via surface receptors CD47 and Integrin α4β1, alleviating atherosclerosis and myocardial infarction. We have drawn a preparation flowchart based on the preparation process of MM/RAPNPs, as shown in Figure 4.

**Figure 5:**
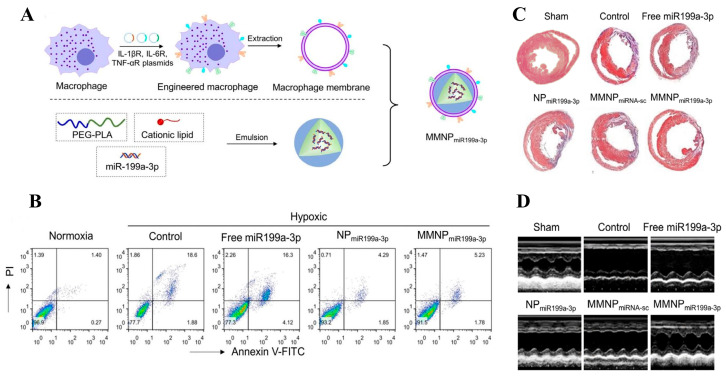
(**A**) Schematic of MMNP preparation process. (**B**) MMNPs inhibit apoptosis of HL-1 cells. (**C**) Masson’s trichrome staining shows MMNPs reduce myocardial fibrosis. (**D**) Echocardiographic results. Adapted with permission from ref. [102]. Copyright © 2020 the authors. All rights reserved.

In the treatment of myocardial infarction, macrophage membrane-modified BNDSs also demonstrate corresponding advantages. For instance, Xue et al. first prepared miR199a-3p-loaded nanoparticles using a double-emulsion method; mPEG5K-b-PLGA11K (25 mg) and BHEM-Chol (2 mg) were dissolved in chloroform, then 200 mg of miR199a-3p was emulsified, and finally mixed with extracted macrophage membrane and extruded to obtain macrophage membrane-coated nanoparticles (MMNPs), as depicted in Figure 5A. To assess the reparative effects of MMNPs on myocardium, HL-1 cells were pretreated with MMNPs for 24 h followed by hypoxic conditions. Results showed hypoxia-induced apoptosis inhibition post-MMNP treatment, as shown in Figure 5B. In vivo experiments further demonstrated that MMNPs reduced myocardial fibrosis area and promoted cardiac function recovery, effectively improving myocardial infarction outcomes, as illustrated in Figure 5C,D. Additionally, the study found that MMNPs possess receptors for IL-1β, IL-6, and TNF-α, enabling binding with these cytokines to suppress inflammatory responses.

#### 5.1.3. Platelet Membranes

Platelets play a crucial role in thrombus formation and maintaining normal vascular morphology [103], making platelet membrane-coated drug delivery systems promising for treating thrombotic and vascular disorders. Researchers have developed a hydrogen peroxide-responsive platelet membrane biomimetic nanosystem for thrombus therapy [104]. Initially, nanoparticles loaded with argatroban (PNPArg) were prepared using nanoprecipitation. Cytotoxicity studies showed PNPArg to be non-toxic to RAW 264.7 cells and HUVECs, enhancing the survival of oxidative stress-damaged cells. Subsequently, a thrombotic mouse model was established to evaluate the pharmacological effects and thrombus-targeting capabilities of PNPArg. Measurement results indicated a thrombus formation degree of 97.8% in the model group, significantly inhibited to 31.8% by PNPArg, markedly better than uncoated platelet membrane nanoparticles (74.5%), demonstrating enhanced targeting by platelet membranes. Glycoproteins on the platelet membrane surface interact specifically with exposed thrombi after vascular injury, facilitating the degradation and release of encapsulated argatroban in the presence of excess hydrogen peroxide. This dual action not only releases the drug but also clears hydrogen peroxide, exhibiting excellent oxygen scavenging capability and thrombus-targeting performance.

While platelet membranes possess some degree of targeting ability, their targeted effect is not long-lasting. Recently, researchers have developed a multi-stage targeted drug delivery system using platelet membrane-loaded arginine and FTY720, achieving sustained targeted delivery [105]. During myocardial ischemia-reperfusion injury, FTY720 targets the lesion area and accumulates in the damaged coronary arteries. Rapid release of arginine stimulates endothelial cells to produce NO, promoting vasodilation and blood flow restoration. In the later stages of reperfusion injury, nanocarriers are captured by inflammatory cells and transported to deeper areas of damage for myocardial repair. This innovative nanocarrier holds significant potential for multi-stage targeted therapy and clinical translation in heart injury.

#### 5.1.4. Stem Cell Membranes

**Figure 6:**
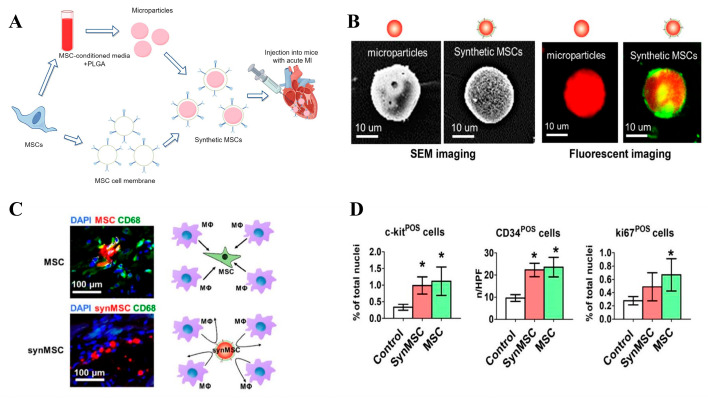
(**A**) Schematic of synMSC synthesis. (**B**) Scanning electron microscopy and fluorescence imaging of synMSC. (**C**) Fluorescence results showing engulfment of MSCs and synMSCs by macrophages after injection into mouse hearts. (**D**) Quantitative expression of positive stem cell markers c-kit, CD34, and ki67. * *p* < 0.05 vs. Control group. Adapted with permission from ref. [108]. Copyright © 2017 the authors. All rights reserved.

Extensive research indicates that mesenchymal stem cells (MSCs) hold great potential in the treatment of CVDs due to their ability to differentiate into mesodermal and non-mesodermal tissues, immunomodulatory properties, and broad-spectrum release of growth factors [106]. MSCs can regulate an overactive immune system as the first line of defense against chronic autoimmune reactions. Leveraging cytokine receptors expressed on stem cells such as CCR1 and CXCR4 enhances MSC migration towards inflammatory and CVD microenvironments [107]. Luo et al. [108] coated MSC membranes onto PLGA nanoparticles (synMSC), as depicted in Figure 6A, characterizing their physicochemical and biological properties in vitro. Scanning electron microscopy and fluorescence imaging confirmed successful membrane encapsulation of nanoparticles, with sizes of approximately 20 μm (Figure 6B). Microparticles were labeled with Texas red succinimidyl ester (red), and synMSC as cell membranes were labeled with green fluorescent DiO (red particle with green coat). Furthermore, they investigated their regenerative potential in a mouse model of acute myocardial infarction. Injection of MSCs and synMSCs into the mouse hearts revealed that MSCs were more prone to engulfment and clearance by macrophages (Figure 6C). Immunostaining analysis of the infarcted area demonstrated that synMSCs promoted proliferation of positive stem cell markers such as c-kit, CD34, and ki67 (Figure 6D), shedding light on the therapeutic mechanisms of synMSCs.

#### 5.1.5. Tumor Cell Membranes

**Figure 7:**
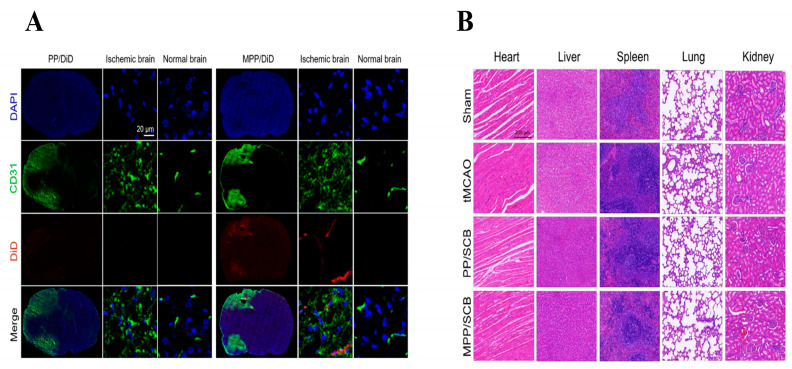
(**A**) In vivo fluorescence distribution of MPP/SCB. (**B**) HE staining of major organs. Adapted with permission from ref. [110]. Copyright © 2021 American Chemical Society. All rights reserved.

Tumor cell membranes contain various types of proteins that can effectively enhance interactions with target cells [109]. These membranes possess unique homologous targeting and immune evasion capabilities, widely applicable in the diagnosis and treatment of tumor-related diseases. Additionally, their abundant expression of functional components provides unique advantages in treating CVDs. He et al. applied breast cancer cell membranes in the treatment of ischemic stroke by constructing biomimetic nanoplatforms loaded with succinate (MPP/SCB) [110]. The expression of syndecan-1 on 4T1 cell membranes promoted blood–brain barrier penetration and enhanced circulation in the cardiovascular region. The transmembrane glycoprotein VCAM-1 on the membrane inhibited endothelial cell adhesion by binding to VLA-1 (α4β1). After labeling MPP/SCB with DiD for fluorescence, its in vivo distribution was measured. The results showed that the fluorescence intensity of MPP/SCB in vivo was significantly higher than that of PP/SCB without cell membrane encapsulation (Figure 7A), indicating the enhanced targeting ability of 4T1 membranes. Organ tissue staining revealed that MPP/SCB effectively improved pathological features and stably exerted therapeutic effects (Figure 7B). The ability of tumor cells to penetrate biological barriers appears promising for targeted therapy in CVDs.

#### 5.1.6. Extracellular Vesicles

**Figure 8:**
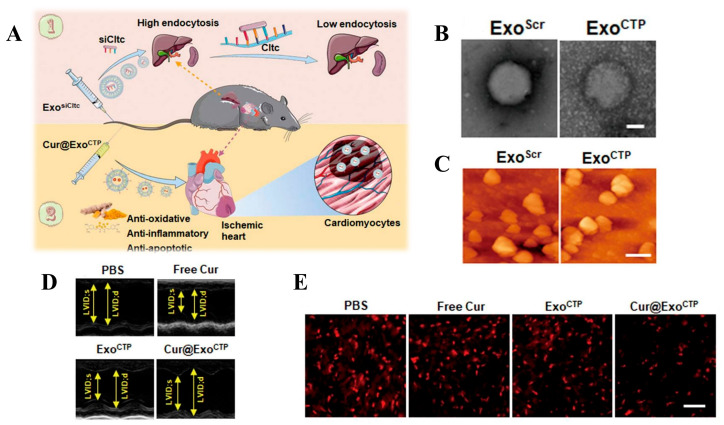
(**A**) Schematic illustration of the study. (**B**) Transmission electron microscopy of Cur@ExoCTP. Scale bar, 50 nm. (**C**) Atomic force microscopy. Scale bar, 200 nm. (**D**) Echocardiography showing Cur@ExoCTP heart function. (**E**) Fluorescence analysis of ROS clearance by Cur@ExoCTP. Scale bar, 200 μm. Adapted with permission from ref. [113]. Copyright © 2023 the authors. All rights reserved.

Extracellular vesicles are membrane-bound vesicles secreted by cells via exocytosis under physiological or pathological conditions, ranging in diameter from 40 to 100 nm and composed of lipid bilayers [111]. Most cells can secrete extracellular vesicles in both normal and pathological states, primarily derived from multivesicular bodies formed by inward budding of lysosomal particles, selectively encapsulating cellular contents such as nucleic acids, proteins, and lipids. Extracellular vesicles exhibit excellent biocompatibility and encapsulation capabilities [112]. Chen et al. reported an exosome-based delivery system targeting curcumin to the heart, effectively alleviating myocardial ischemic injury [113]. Extracellular vesicles isolated from conditioned media of cultured bone marrow mesenchymal stem cells were mixed with peptides, followed by curcumin dissolved in DMSO to obtain Cur@ExoCTP (Figure 8A). Transmission electron microscopy and atomic force microscopy revealed spherical and well-dispersed structures (Figure 8B,C). Evaluation of Cur@ExoCTP in mice induced with myocardial infarction showed significant improvement in cardiac function parameters at days 0, 1, 2, 7, 14, and 28 post-surgery (Figure 8D). Additionally, Cur@ExoCTP mitigated excessive ROS accumulation induced by myocardial infarction (Figure 8E), effectively modulating levels of malondialdehyde, lactate dehydrogenase, and superoxide dismutase to alleviate oxidative stress.

Additionally, Zhao et al. developed MSC-Exo, a nanoliposome modified with extracellular vesicles, which promotes macrophage M1-to-M2 polarization, reduces inflammation, and protects cardiomyocytes [114]. Injection of MSC-Exo into myocardium inhibits myocardial fibrosis, improves cardiac function, and reduces infiltration of inflammatory cells. Measurement of pro-inflammatory cytokines IL-6 and IL-10 in serum and myocardial tissue post-surgery reveals elevated levels in the model group, whereas treatment with MSC-Exo significantly lowers IL-6 and IL-10 levels. Interestingly, Zhang et al. prepared DDN, a danshen-derived exosome nanoparticle, with an average size of 105.15 nm and a zeta potential of −25.2 mV, demonstrating good stability and storage properties after centrifugation purification [115]. In vitro experiments show that DDN enhances HUVEC viability and promotes cell proliferation, and scratch assays confirm its ability to facilitate cell migration. Pathological tissue examination indicates that DDN improves myocardial tissue fibrosis, maintains vascular morphology, and causes no pathological damage to the liver, spleen, lungs, or kidneys.

Interestingly, the size of extracellular vesicles is similar to that of constructed nanodelivery systems, allowing them to serve as standalone carriers for drug delivery. BNDSs, with drugs encapsulated in biomembranes, can leverage surface characteristics and functional modifications to achieve higher targeting specificity and controlled drug release [116]. Conversely, direct encapsulation of drugs within extracellular vesicles takes advantage of their natural cell-to-cell communication capabilities and excellent biocompatibility. The choice between these two methods in practical applications may depend on specific therapeutic needs and the characteristics of the target disease. However, relevant studies have demonstrated that modifying extracellular vesicles into nanodrugs enhances their therapeutic potential. Li et al. [117] conducted comparative experiments and found that drug-loaded, nanoparticle-modified extracellular vesicles exhibited superior therapeutic effects compared to natural extracellular vesicles, effectively applying to ischemia-reperfusion injury.

#### 5.1.7. Composite Hybrid Membranes

Various types of biomembranes possess specific characteristics. Therefore, in addition to constructing BNDSs using single biomembranes, research increasingly focuses on combining two types of biomembranes to form composite BNDSs. Such hybrid membranes aim to amplify the advantages of both cell sources. Huang et al. combined red blood cell membranes with platelet membranes to simulate two cellular sources in vivo, aiming to prolong drug circulation [118]. They mixed red blood cell membranes and platelet membranes in a 1:1 ratio, then incorporated PLGA nanoparticles containing the antibody drug CXCR2 to form NPs-CXCR2. Encapsulation efficiency of NPs-CXCR2 was found to be approximately 80%, with a high drug loading of up to 20% and a particle size of 90 nm. Stability evaluation of NPs-CXCR2 over 20 days in PBS and fetal bovine serum showed minimal changes in particle size and absorbance after freeze–thaw cycles, indicating excellent stability. Targeting assessment in an atherosclerosis mouse model demonstrated preferential accumulation of the drug in the liver and spleen.

**Figure 9:**
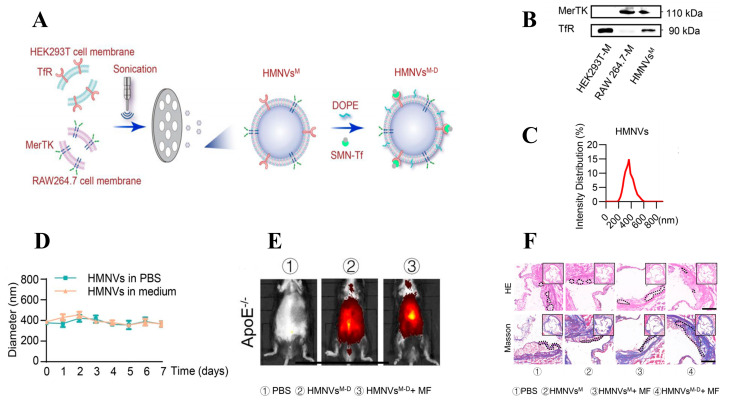
(**A**) Schematic illustration for the preparation of HMNVs. (**B**) Western blot analysis of MerTK and TfR expression in HMNVs. (**C**) Size distribution of HMNVs. (**D**) Particle size change in HMNVs in PBS and medium. (**E**) Representative in vivo imaging system images of mice and aorta injected with HMNVs via tail vein. (**F**) Representative images of the atherogenic lesion area stained with hematoxylin and eosin (H&E), Masson’s trichrome. Adapted with permission from ref. [117]. Copyright © 2024 the authors. All rights reserved.

In contrast to biomimetic drug loading using red blood cells and platelets, Qiu et al. [119] focused on the anti-inflammatory effects of macrophages. They mixed cell membranes overexpressing transferrin receptor (TfR) from RAW264.7 and HEK293T cells with DOPE polymer to create nano-vesicles named HMNVs (Figure 9A). HMNVs can fuse with receptor cell membranes, enhancing macrophage anti-inflammatory effects and restoring iron phagocytic capacity. MerTK is a critical target in diabetes-related arterial atherosclerosis; inhibiting MerTK damages macrophages, leading to apoptotic cell accumulation and atherosclerotic lesions. MerTK protein was detected on HMNVs they prepared (Figure 9B), indicating potential targeting efficacy at plaque sites. Nanoparticle tracking analysis showed uniform size distribution of HMNVs, with diameters between 300 and 400 nm (Figure 9C). Furthermore, HMNVs exhibited good size stability in PBS and DMEM over 7 days (Figure 9D). DiO/DiR-labeled HMNVs were intravenously injected into ApoE^−/−^ mice, and live imaging analysis revealed increased HMNVs in the aorta (Figure 9E), with significantly higher fluorescence intensity than controls, indicating prolonged circulation time of drugs encapsulated under hybrid membranes. Histological staining of aortic tissues with HE and Masson staining showed varying degrees of improvement following treatment with each nanomedicine (Figure 9F). Hybrid membrane-encapsulated nano-vesicles appear promising for targeted drug delivery against arterial atherosclerosis.

### 5.2. Imaging and Diagnosis

At the forefront of research and treatment for CVDs, diagnostic and imaging techniques are crucial. BNDS, owing to its unique physicochemical properties and biocompatibility, is rapidly becoming a key tool in this field. For predictive diagnostics in CVDs, imaging techniques combined with targeted contrast agents are essential [120,121]. Contrast agents enhance the visibility of lesion detection images, thereby improving molecular imaging sensitivity and specificity [122]. Therefore, the primary requirement for imaging is the selection of reliable contrast agents. Ma et al. [123] designed a lipid-specific fluorescent probe that, together with the reactive oxygen species-responsive drug prednisolone, forms nano-micelles, further coated with red blood cell membrane to form biomimetic nano-micelles (RBC/LFP@PMMP). Characterization revealed that RBC/LFP@PMMP extends drug release duration and exhibits low drug leakage. Due to the reactivity towards ROS, RBC/LFP@PMMP can target and release the fluorescent probe binding to lipids in inflamed atherosclerotic tissues overexpressing ROS, enabling precise anti-inflammatory and lipid-specific fluorescence imaging of atherosclerotic lesions.

**Figure 10:**
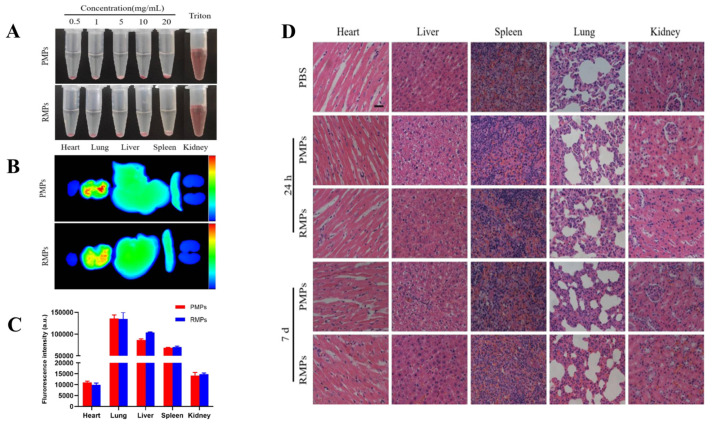
(**A**) Hemolysis effects on RBCs of PMPs and RMPs. (**B**) Representative ex vivo fluorescent images of major organs. (**C**) Quantitative analysis of fluorescent intensities. (**D**) H&E staining of major organs after 24 h and 7 d of PBS, PMP, or RMP administration. Scale bar, 20 μm. Adapted with permission from ref. [125]. Copyright © 2021 American Chemical Society. All rights reserved.

Nanomaterials offer unique advantages for imaging, considering several factors. First, biomembranes provide large surface areas capable of accommodating more contrast agents [124]. Their structural specificity allows targeted imaging of diseases through surface functionalization. Controlled drug release in response to specific stimuli reduces off-target effects and enhances therapeutic efficacy [125]. Early diagnosis of myocardial ischemia-reperfusion injury is crucial for myocardial protection and patient prognosis improvement [126]. Platelet membranes possess the ability to target injured areas, facilitating early detection using PLGA nanoparticles for ultrasound imaging [127]. Researchers prepared biomimetic nanoparticles wrapped with platelet membranes (RMPs) and control nanoparticles without membrane wrapping (PMPs), confirming the membrane coatings of PMPs and RMPs through transmission electron microscopy and fluorescence microscopy. In vitro hemolysis tests demonstrated good biocompatibility of RMPs without hemolytic effects (Figure 10A). In vivo fluorescence imaging showed superior targeting capability of RMPs (Figure 10B,C), accumulating predominantly in the lungs. HE staining indicated better improvement in myocardial tissue with RMPs, reducing myocardial fibrosis (Figure 10D). In conclusion, this ultrasound imaging approach enables non-invasive detection of early myocardial injury.

In addition, nanoprobes have emerged as a hot topic in CVD imaging and diagnostics. Biomimetic nanoprobes, integrating biomimetic and nanotechnological approaches, significantly enhance the precision and efficiency of imaging diagnostics by mimicking natural molecules or structures within the body [128,129]. These probes typically consist of nanomaterials combined with biomolecules, enabling specific targeting of cardiovascular lesions and providing high-resolution images across multiple imaging modalities [130]. Biomimetic nanoprobes are nano-formulations surface-modified with biomolecules such as antibodies, peptides, and sugars [131]. Leveraging the specific recognition capabilities of these biomolecules, they precisely bind to specific targets associated with CVDs, commonly used in various imaging techniques including magnetic resonance imaging (MRI) [132], computed tomography (CT) [133], positron emission tomography (PET) [134], and optical imaging [135]. For instance, Fracassi et al. developed lipid nanoparticles (LNPs) with a hydrophobic core encapsulating drugs and imaging probes to create highly efficient MRI probes [136], enhancing atherosclerotic plaque visibility in mouse models, and observed their presence through high-emission fluorescence imaging. Ma et al. utilized the close relationship between oxidative stress and atherosclerosis to design a photoacoustic imaging diagnostic tool, reflecting plaque burden and oxidative stress levels [137].

## 6. Conclusions and Perspectives

Since the concept of BNDS was introduced, extensive research has emerged exploring its potential in diagnosing and treating CVDs. As reviewed, BNDSs can effectively reduce immune recognition and clearance, enhance drug biocompatibility and circulation time, significantly improve targeting, and ensure precise delivery to diseased sites, enabling multifunctional therapy.

Significant progress has been made in BNDS research. By utilizing various biomembranes, such as erythrocyte membranes, macrophage membranes, platelet membranes, stem cell membranes, and tumor cell membranes, researchers have successfully constructed diverse drug delivery systems. These systems have demonstrated effective drug delivery and biocompatibility in experimental and preliminary clinical trials. BNDSs effectively deliver therapeutic agents for CVDs, such as anticoagulants, lipid-lowering drugs, and antihypertensives, by enhancing drug stability and half-life in circulation, thus achieving sustained release and therapeutic effects [138,139]. For CVD diagnosis and imaging, biomimetic nanoprobe technology enables high-resolution and high-specificity imaging, facilitating precise identification and localization of cardiovascular lesions for early diagnosis and targeted therapy [140]. Although clinical applications of BNDSs are still in the early stages, promising research outcomes suggest considerable potential. For example, drug delivery systems based on stem cell membranes have shown significant therapeutic effects in animal models [141], indicating broad prospects for future clinical applications.

Despite significant advancements in the treatment of CVDs using BNDSs, numerous challenges and bottlenecks persist in their research and application. The long-term biocompatibility and safety of BNDSs modified with biological membranes in vivo require further validation. The toxicity of BNDSs primarily arises from two sources: the inherent toxicity of the materials and the potential adverse reactions from their metabolic and degradation products within the body. For the former, different materials, such as PLGA, liposomes, and metal nanoparticles, exhibit varying degrees of biocompatibility in vivo, necessitating acute and long-term toxicity assessments through both in vitro and in vivo experiments. For the latter, the degradation products of BNDSs in vivo may trigger immune or inflammatory responses, necessitating precise chemical modifications and encapsulation techniques to mitigate toxicity. The intrinsic properties of nanomaterials, such as size, shape, and surface characteristics, can influence their distribution, metabolism, and excretion processes within the body. Certain nanoparticles may persist in the body for extended periods, leading to potential toxic reactions. The source and modification methods of biological membranes also impact their biocompatibility and safety. For instance, biological membranes from different sources may possess varying immunogenicity, resulting in different degrees of immune responses. These issues remain critical challenges in current research, requiring extensive experiments and clinical trials to evaluate their safety for long-term use.

Due to their unique biocompatibility and functional properties, BNDSs demonstrate great potential in drug delivery. However, their drug release mechanisms are complex and include pathways such as natural degradation, external stimulus response, cellular phagocytosis, and lysosomal escape. Once inside the body, the biomembrane component of the BNDS gradually degrades, slowly releasing the encapsulated drug. This degradation process depends on the composition of the cell membrane and its interaction with the internal environment. Some BNDSs are designed to respond to specific external stimuli (such as pH, temperature, or light) to control drug release. Additionally, when BNDSs target the disease site and enter the lysosomal environment of cells, the membrane structure is disrupted, allowing the drug to be released. The composition and structure of different biomembranes affect their stability and degradation rate in vivo. For instance, red blood cell membranes are relatively stable and degrade slowly, while tumor cell membranes, which contain more proteins and carbohydrates, may degrade more quickly. The size and surface modifications of nanomedicines also significantly impact the drug release rate. Smaller particles typically have a larger surface area, potentially leading to a faster release rate, whereas surface modifications can provide an additional barrier to delay drug release. Therefore, it is crucial to optimize the source of cell membranes and the design of the delivery system for different diseases to better predict and control drug release behavior.

The preparation of BNDSs is relatively complex, and achieving scalable production with consistent quality is crucial for clinical application. BNDS preparation involves multi-step processes, including nanomaterial synthesis, biomembrane extraction and modification, and drug encapsulation and loading. These steps are time-consuming and labor-intensive, requiring stringent control conditions to ensure effectiveness and reproducibility. Biomembrane extraction and modification are key steps, involving cell disruption, centrifugation, and filtration, each affecting the purity and functionality of the final product. Different sources of biomembranes have varying extraction complexities. For example, erythrocyte membrane extraction is relatively simple, while stem cell membrane extraction requires more precise operations and equipment. Consistent particle size, morphology, and functionality in large-scale production demand high stability and reproducibility in the process, necessitating rigorous preparation techniques to ensure product stability.

Furthermore, the targeting efficiency and therapeutic efficacy of BNDSs in practical applications require substantial optimization. Biological barriers and nonspecific adsorption during circulation can significantly affect the targeting ability of nanocarriers. Factors such as hemodynamics, immune clearance mechanisms, and nanocarrier–cell interactions play crucial roles in influencing targeting efficiency. The diverse pathophysiological states of different patients necessitate highly personalized and adaptable nanodrug delivery systems to meet varying therapeutic needs. Enhancing the accumulation and retention time of nanocarriers at diseased sites while minimizing off-target drug release remains a critical research focus. Translating from laboratory research to clinical applications, BNDSs face numerous challenges, including biocompatibility, safety, scalability of production, drug release control, clinical trial design, and market entry.

Facing these challenges offers new insights and development opportunities. To ensure the safety of BNDSs, it is imperative to assess the biocompatibility and toxicity of various nanomaterials and biomembranes early on, especially for long-term use. Additionally, developing novel biomembranes and nanomaterials can further enhance the performance of biomimetic nanodrug delivery systems. For instance, genetic engineering can modify biomembranes to improve their targeting and biocompatibility. Chen et al. [142] developed a nanoparticle coated with bacterial membranes that overcomes the blood–brain barrier via transcellular vesicle transport, providing extended circulation time for drugs. Krishnan et al. [143] explored the introduction of additional functionalities to enhance BNDS performance, using genetically engineered protein receptors, aptamers, and single-chain variable fragments to modify nanoparticles. These modified particles exhibited significant affinity for cell lines with overexpressed ligand homologous receptors and showed strong targeting and growth inhibition in a mouse ovarian cancer xenograft model. For scalable production, future research should focus on developing simpler and more efficient preparation processes, as well as exploring more stable and reproducible biomembrane extraction methods to ensure consistent quality and functionality in large-scale production. Establishing stringent quality control systems with standardized processes and testing methods is crucial to ensure batch-to-batch stability and safety. Multifunctional nanoplatforms are also a promising target, combining diagnosis, therapy, and monitoring into an integrated treatment approach. Liu et al. [144] proposed a multifunctional nanoplatform based on hyaluronic acid modifications that integrates starvation therapy (ST), chemodynamic therapy (CDT), and photothermal therapy (PTT) for targeted cancer treatment, effectively inhibiting tumor growth while providing cancer imaging. Such multifunctional platforms also offer valuable insights for CVD treatment.

In conclusion, research advancements in BNDSs for CVD treatment offer a novel therapeutic strategy. As research deepens and technology advances, this field holds promising prospects, providing more effective and safer treatment options for CVD patients. Through multidisciplinary collaboration, BNDSs will play a crucial role in the future diagnosis and treatment of CVDs, promoting the development of cardiovascular medicine towards precision, personalization, and integration.

## Figures and Tables

**Table 1:** Types and characteristics of biomembranes in BNDS.

Type	Advantages	Disadvantages	Refs.
Erythrocyte membranes	Easily obtainable, long circulation time, low immunogenicity, high biocompatibility	Considerations for blood type compatibility	[62]
Macrophage membranes	High biocompatibility, low toxicity, inflammation targeting	Complex preparation, potential for immune reactions	[63]
Platelet membranes	Inflammation targeting	Complex preparation, restricted storage conditions	[64]
Stem cell membranes	Low immunogenicity, promotes tissue regeneration and functional recovery	Limited source, potential for heterogeneity	[65]
Tumor cell membranes	Tumor targeting	Biological safety remains unclear	[66]
Extracellular vesicles	High biocompatibility, low immunogenicity	Complex preparation	[67]
Composite hybrid membranes	High biocompatibility, high targeting	Low stability	[68]

**Table 2:** Methods and characteristics for extraction of biomembranes.

Method	Principle	Advantages	Disadvantages	Applications	Refs.
Ultrasonication	Using ultrasonic energy to disrupt cell membrane structure and release cytoplasm containing membranes	Efficient and straightforward	Excessive acoustic power may compromise membrane structure	Wang et al. found that ultrasonic extraction rapidly disrupts keratin biomembranes, demonstrating its efficiency and convenience	[79]
Centrifugation	Cell membrane-containing supernatant was separated from other cellular components using varying centrifugation speeds and times	High purity and broad applicability	Significant loss	Qing et al. used centrifugation to extract red blood cell membranes for nanoparticle drug delivery. Hemoglobin was effectively removed through hypotonic centrifugation	[80]
Freeze–thaw cycle	By repeatedly freezing and thawing cell samples, the freeze–thaw process induces volume changes in water, disrupting cell membranes and releasing membrane structures	Efficient and straightforward	Low purity	Ivan et al. found that slow freeze–thaw cycles can eliminate residuals in red blood cell membranes, and hemoglobin denatures at 49 °C	[81]
Chemical approach	Disruption of lipid bilayer structure of cell membranes using surfactants or solvents to release membrane lipids and proteins	High purity and strong controllability	High cost and residual impurities	John et al. used chemical detergents to rapidly detach proteins and obtain pure cell membranes	[82]

**Table 3:** Different types and applications of BNDSs.

Type	Characteristics	Applications	Refs.
Nanoparticles	The drug and carrier form solid colloidal substances with particle sizes ranging from 10 to 1000 nm, characterized by high surface area and high drug loading efficiency	Insu Kim et al. prepared erythrocyte membrane-functionalized Au nanoparticles for rapid fibrinogen detection via receptor cross-linking, aimed at CVD diagnosis	[93]
Nanoliposomes	Nanoscale vesicles formed by phospholipid bilayers, offering high biocompatibility, drug encapsulation, and targeted delivery advantages	Liu et al. developed a macrophage membrane-coated liposome co-loaded with Panax notoginseng saponins and ginsenoside Rg3 using Box–Behnken design for targeted therapy of ischemic stroke	[94]
Nanomembranes	Nanostructures formed by self-assembly of surfactants, exhibiting high drug loading capacity and good biocompatibility	Shi et al. developed nanomicelles using quercetin and polyethylene glycol, finding they can alleviate atherosclerosis by modulating gut microbiota composition	[95]
Nanoemulsion	Nano-emulsion composed of oil phase, water phase, and emulsifier, exhibiting high stability and bioavailability	Anghelache et al. developed a novel carrier, Bio-LN/SPMs, by coating macrophage membranes onto lipid emulsions containing lipolytic mediators. Their study demonstrated reduced lipid accumulation and inflammation levels in a mouse model of aortic disease.	[96]
Nano-alcohol plasma	Nanodelivery structures composed of phospholipids and high concentrations of ethanol, exhibiting high permeability and drug loading capacity	Liao et al. formulated ellagic acid with 30% ethanol and 1% Tween-80 into nano-cochleates. Transmission electron microscopy revealed that the nano-cochleates exhibited a compact and intact morphology. In vitro experiments also demonstrated their potential for transdermal drug delivery.	[97]
